# A Treatise on the Plague, Designed to Prove It Contagious, &c.

**Published:** 1821-03-01

**Authors:** 


*A Treatise on the Plague, designed to prove it contagious, from Facts, collected during the Author's Residence in Malta, when visited by that Malady in* 1813. *With Observations on its Prevention, Character, and Treatment; to which is annexed an Appendix, containing Minutes of the Author's Evidence, given before the Contagion Committee of the House of Commons, accompanied by their Report.* By Sir ARTHUR BROOKE FAULKNER, M. D. Fellow of the Royal College of Physicians; late Physician to His Majesty's Forces, and Physician in Ordinary to His Royal Highness the Duke of Sussex. One vol. octavo, pp. 312, with two plans. London, 1820.

---

"Mittant pestiferos Œstus et tetra venena." *Val. C.*

"PESTIS, et ira Deum Stygiis sese extulit undis." *Virg.*

---

IT hath pleased the ALMIGHTY (for we are yet superstitious enough to believe in the existence of a DEITY) to permit good and evil to be promiscuously scattered through every people on the earth, and through every class of society. It is equally wonderful and fortunate that every nation or tribe becomes firmly attached to its natal soil, however barren, or however baneful that soil may be! No consideration can draw the Hollander from his noxious fens—nor the Swiss from his naked mountains. The Negro basks with delight in the scorching ray of a tropical sun—the Russian rolls himself in the chilling snow. The Byzentine fatalist calmly inhales the pestilent breath of contagion—the Briton sees his family and friends cut off in succession by the gloomy climate. Yet all cling to the land that gave them birth:—

"And each proclaims that happiest clime his own."

This is a universal propensity of Nature; for animals possess it in the strongest degree—next to them the savage—

then the half-lettered peasant—and last of all the cosmopolitan philosopher. How is this to be accounted for? There is implanted a sense of gratitude (whatever may be said to the contrary) in the breast of all animated beings. The earth that feeds them, the house that shelters them from their infancy, become at length endeared to them, and preferred to better, but less familiar abodes.

These, among other reflections, have been excited by the perusal of an interesting topographical sketch of the island of Malta, with which Sir Brooke Faulkner opens the work before us, and of which we shall endeavour to convey some idea to our readers.

Were it not for the rude and unsightly interference of quarries and stone ditches, Malta would present a pleasing aspect; as there is hardly a foot of the rock, with a covering of mould, that is not laid under contribution for the production of corn, cotton, fruits or roots, every season yielding a more abundant crop, in proportion, than Sicily itself, the once famed granary of Rome. The face of the country, however, when viewed in perspective, resembles an extensive stone-cutter's yard—a great part of the island, especially to the westward, being a barren-waste, "presenting little beside the bare bones of Nature, which, in all probability, will never be clothed." Sir Brooke has gone into these details respecting the surface of the soil, in order that the advocates for the generation of plague, by noxious exhalation from the ground, may see how far they are deprived of the benefit of such an hypothesis, when applied to such a country as Malta, and to such a city as Valetta, which we shall presently describe. There are, however, three spots on the habitable part of the rock, which may be termed marshy or moist; and our author endeavours, in another part of the work, to prove, that these places exerted no kind of influence in producing the plague, being not only remote from the towns which were first assailed, but having indeed very few cases of the disease in their vicinity. There are neither rivers, plantations, or morasses, in any part of the island.

Although the climate of Malta is hot, it has many other advantages, there being rarely any fogs or vapours—and excepting in the first three months of the year, the sky is one continued azure, with seldom a cloud to intercept the rays of the sun. In summer the thermometer ranges from 75° to 85°, in the shade. Owing, however, to a deception of the sense, the oppressive languor caused by the Sciroc wind, imparts a sensation of the climate being much warmer than the thermometer indicates. Sudden atmospherical transitions, from heat to cold, are felt in a remarkable degree; when the wind shifts from a southern to a northern point—a circumstance common to most parts of the Mediterranean near the African coast. And, *e contra*, when the wind suddenly changes from North to South-East, the rise in the temperature is frequently twenty degrees.

These vicissitudes of temperature rarely produce any serious detriment to the Maltese constitution. "The most delicate habits will emerge from three or four days of Sciroc, with as much impunity as if the temperature had been perfectly uniform and moderate." The change from Sciroc imparts, of course, a feeling of elasticity and exhilaration, resembling that which follows the cold bath in sultry weather. The effects of the Sciroc are evidently something more than thermometrical; but what the peculiar quality of this wind is, we at present know not.

The seasons of one year do not differ materially from those of another—there is not, indeed, much variable weather, excepting in early spring, when rain falls in considerable quantities, and tempestuous gales upset the small craft in the harbours. These are the Euroclydons which proved so disastrous to St. Paul. Snow and ice are seldom seen, and, when seen, are very transient. In hot summer weather the night is the most oppressive part of the twenty-four hours, being nearly as hot as the day. On this account the dews are necessarily inconsiderable.

"Thunder and lightning are not unfrequent occurrences; nor can any thing be imagined more awfully magnificent than the sky, dressed in all its gloomy terrors, whilst the vivid flashes dart across the horizon in rapid succession. The thunder-peal is truly terrific, and has often brought to my recollection the following passage, in which its effects are described with such majesty:—

> 'Quo bruta tellus, et vaga flumina

> 'Quo Styx, et invisi horrida Tænari

> 'Sedes, Atlanteusque finis

> 'Concutitur————'

On these occasions the rain falls in torrents, but is soon over, and the sky resumes its natural serenity." 15.

There are about twenty-four small towns, or *casals*, on the island, all of which are built of hewn stone, and are both commodious and healthy. Between these are sprinkled a few country-houses and hamlets.

"The natives are healthy, active, strong, industrious, and sober; and so devoted to their country, as to think no other on the face of the earth can come into comparison with it. Hence they dignify it with the title of 'Fiore del Mondo.' Their diet is simple, consisting of vegetables, maccaroni, fruit, eggs, oil, cheese, honey, and a little animal food, (for the most part fish,) and they are very temperate in the use of wine and spirits." 16.

The adjacent island of Gozo, though standing higher, has a leveller surface, gives less trouble to the husbandman, and makes a better return to his labour. Malta is about twenty miles in length by twelve in breadth. Gozo nine by five.

From the above topographical sketch of these islands, our author concludes, that "there is no *prima facie* evidence of their being liable to generate pestiferous miasmata." Of this position we entertain some doubts. We fear there are few spots of the earth so favoured as not to give origin, under peculiar circumstances, to morbific effluvia. But what these peculiar circumstances are, we cannot yet ascertain. Neither does this event at all affect the object which our able author labours to attain.

The second chapter of Sir Brooke's work being argumentative on the "chief causes which have led to errors, respecting contagion," we shall pass it over, as not adapted to analysis, though containing many shrewd and excellent observations. The third chapter adduces "facts, in proof of the contagious nature of the plague." These are arranged under three heads. 1st. The extension of the disease to Valetta from an infected vessel, the San Nicholo. 2d. Its extension to individuals who were infected by communicating with the first case in Valetta—and to the Augustin convent. 3d. Its extension to certain casals, and to the Island of Gozo.

The first position, or the introduction of the contagion from the vessel to the shore, our author fails to prove by *direct* evidence. The current report at Malta was, that one Salvator Borg, a shoemaker, having purchased a piece of linen on board the San Nicholo, introduced the fomites into his own family, whence it radiated afterwards to other persons. Dr. Granville has since stated that this was authenticated as a fact, under the authority of Sir Thomas Maitland's dispatches, and other official papers.

"It may be satisfactory to the reader to be informed of the following particulars relating to the arrival of the San Nicolo:

Two Turkey merchants shipped on board this vessel, at the Port of Alexandria, a cargo of linen, flax, and leather, with some other articles. Part of the crew having died of the Plague on their voyage to Malta, the vessel applied to the Health Department of the Island on her arrival (the 28th March) for admittance into Port, previously using the precaution to notify her state, by hoisting a yellow flag with a black ball in the centre, this being the signal to indicate the actual existence of Plague on board. Her application being acceded to, she was accordingly received into quarantine in the Marsamuchet Harbour, within about a cable's length of several points of land, and of the city of Valletta. The surviving part of the crew were taken into the Lazaretto, situated in a small island in the middle of the harbour. The captain of the San Nicolo and his servant sickened in a day or two after their being received into the Lazaretto, and died with indisputable symptoms of the Plague." 46.

In a few days after this occurrence, and after an immunity from plague of 137 years, this dreadful scourge manifested itself in Valetta. The first person attacked was Salvator Borg's daughter, residing near the centre of Strada St. Paulo, one of the principal streets of Valetta. This was on the 16th of April. She was visited on the 19th by a Maltese physician, who found her in a dying state. She died on the same evening. He observed two tumours on her chest, below the mammæ, resembling carbuncles. The mother, then pregnant, was seized on the 1st May, and died on the 3d, with buboes in both groins. On the 4th May, her husband, Salvator Borg, was attacked with fever, having at the same time the glands of the axilla and groin enlarged. He lingered till the 12th, when he expired with unequivocal symptoms of plague. The next victim, in succession, was Maria Agius, a school-mistress, who was a frequent visiter in Borg's family, and who assisted his wife, on her death-bed. This woman died on the 6th May, "with unquestionable marks of the disease." On the 8th May, Grazia Pisani, a girl on terms of the closest intimacy with Agius, was seized with the disease, but recovered. On the above day also, Salvator's father was attacked, and died the next; on the 14th another child of Salvator's was seized with plague, and ultimately died. On the 17th, Arcangelo Delivato, after a residence of eleven days under observation, in consequence of having held communication with Maria Agius, became affected with well-marked symptoms of the disease, and sunk the next day with a carbuncle near the os sacrum. This man was accustomed to visit Agius before and after her attack.

"Here, then, we have traced the propagation of the disease from the first case in Valetta, *in eight distinct and well authenticated instances*, and all of them in the continuous line of communication with each other. The last six cases are given on the authority of Medical Reports, published under sanction of the Government of Malta." 65.

About this time the contagion, our author informs us, began to diverge in so many directions, in consequence of the unrestrained intercourse of the people, that it would have been extremely difficult, if not impracticable, to trace the line of contamination, which, however, was never earnestly pursued. To this difficulty there was one exception, namely, the Augustin Convent, which, seated in a peculiarly healthful, spacious, and airy part of Valetta, had, from the very beginning, observed the greatest caution in shunning communication with the public.

"It was at length, however, infected, in consequence of one of the servants, who was caterer by occupation, having, in disobedience of public orders, gone into a very contaminated part of the town, called the Manderaggio, and purchased infected clothes. Shortly after his return he made full confession of the circumstance, when one of the brotherhood belonging to this convent, out of compassion, immediately volunteered to attend him, placing himself, at the same time, in strict quarantine with the patient. Both nurse and patient immediately fell victims to the disease, but *no other individual under the same roof was ever assailed.*" 68-9.

Judging from the advantageous situation of the convent, in point of salubrity, "it was perhaps the very last place where ingenuity could discover a pretext to explain the production of the disease through the agency of any other than a contagious cause." Cavallino, an historian of the plague which visited Malta in 1675, observes, that all public buildings which cautiously shunned intercourse with the community, enjoyed a perfect exemption from the contagion, among which were the prisons, monasteries, and the CAVALIERI occupied by the Knights of Malta.

In the second section our author traces the introduction of the plague into the casals, or inland towns and villages, and that on evidence guaranteed by written documents, supplied him by one of the captains of the Port of Malta, the nature of whose services afforded him peculiar facilities of procuring information on the subject of pestilential contagion.

"It is this officer's opinion, that wherever the plague makes its appearance, and proper measures are strictly enforced, *sur le champ*, its progress may be completely arrested, not only within the limits of the infected families, but may be prevented from extending even beyond the *individual attacked in the very first instance*, provided he be instantly removed out of the reach of communicating with others, and that the susceptible articles with which he has been in contact, undergo a thorough expurgation. The truth of this has been repeatedly exemplified during the late sufferings of Malta, the disease being arrested at once in several of the casals, in the houses originally contaminated, by the immediate adoption of rigorous and prompt measures of separation and seclusion, under the direction of the magistrates. In other casals, where these measures were either delayed, or were not so decidedly acted upon, the Plague spread to an alarming extent." 74.

For the contents of this section, and the whole of the fourth chapter, in which farther evidence in proof of the contagious property of the plague is drawn from the extent of contamination in each of the casals, we must refer to the work itself, in which the reader will find important facts and powerful arguments.

In the 5th Chapter our author brings forward strong proofs that an impure state of the atmosphere, filth, crowding, and want of ventilation are, of themselves, insufficient to account for the generation of the plague. We can only glance at a few of these proofs. The casal Zebbuy, for instance, was ravaged to an extent far exceeding every other, though it is one of the most favoured spots, in point of locality, cleanliness, and ventilation, of any in Malta. On the other hand, the 14th Regiment and Artillery, quartered in one of the most infected parts of Valetta, and surrounded by a crowded neighbourhood, were preserved from the contagion throughout the whole period of the disease, by the early restrictions imposed by their commanding officers, and an unrelaxing vigilance to interdict every kind of intercourse with the public. But our author does not deny that an impure atmosphere, closeness, crowding, and filth, may powerfully predispose to render pestilential contagion more active, as they favour the progress and aggravate the malignity of other contagious diseases; he only argues that the specific contagion must also be present.

From accurate thermometrical tables, our author shews that *temperature* had little or nothing to do with the increment or decrement of the plague at Malta, since it was remarked that the thermometer stood at 76$^{d}$ when the last case was announced in Valetta, which was a degree of heat equal to that in which the contagion made its most rapid strides, at an earlier period, in the same city.

"Finally;" says our author, "when to the whole of this body of facts we add the rapid diminution in the number of attacks following almost immediately upon the rigid enforcement of Police restrictions in the several towns formerly mentioned, and at a period when the contagion has attained to its acmé of diffusibility, no doubt, I think, can remain in any mind which is open to conviction, that it was to these measures alone the sudden cessation ot the disease in those places was immediately to be attributed, and not to any alteration in the atmospheric influence." 177.

Our author denies the validity of the commonly received opinion, that the plague arises and disappears at certain determinate periods of the year—"an assertion which is unsupported by fact, as the disease is known to commence in the same country under every diversity as to the seasons." He instances the last two plagues at Malta, one of which commenced in December, three months previous to the appearance of the other.

### PREVENTION OF THE PLAGUE.

"That as man continues, age after age, to disregard the lessons of experience, there is but a slender hope of his condition being materially improved, was the reflection of a profound observer;* and the force of it was indeed most fatally exemplified during the late Plague of Malta. Upon that unhappy occasion, the vigorous precautions, long sanctioned by the practice of mankind, and repeatedly proved to afford the only chance of protection against this insidious disease, were so tardily, or imperfectly carried into execution, that it became impossible to put an immediate stop to its progress." 197.

Long after the disease was detected in Valetta, on the late occasion, the decrees of public health were exclusively taken up with merely *recommending* measures of secondary importance, in place of peremptorily requiring obedience to those upon which the efficacy of every other depended!

"———————— to their decrees
"Dead to infliction, to themselves were dead." P. 200.

After a tiresome, ridiculous, and fatal system of trifling and procrastination, an effective police was at length instituted, on the third July, and the measures adopted were, *segregation and seclusion*, almost identically the same as were recommended by our author on the first appearance of the plague, the 5th of May preceding. These means were, in a very short time, followed by the most visible success; for on the 17th July, it was proclaimed that the contagion had received a decided check. The people were absolutely *incarcerated* in their houses, with the exception of the public officers, a measure that, on first proposal, was thought to be impracticable, till the experiment was tried in a single district and found to be perfectly successful. At this time no one was permitted in the streets, under pain of capital punishment. The same measures were extended to the casals. Provisions and necessaries of all kinds were carried to the houses of the inhabitants, and no shops were allowed to be open—in short, the people were, bona fide, in street quarrantine, and to intimidate the refractory, a Maltese, who was detected concealing his illness, was publickly shot!

The 7th Chapter is on the symptoms, treatment, &c. of the plague at Malta; and here our author regrets that the experience of the medical authorities has been productive of but little accession to our knowledge, in respect either to the rationale or treatment. Our author's symptomatology of the disorder we shall pass over, as it was substantially published

* Volney.

some years ago in our respected cotemporary, the Edinburgh Medical and Surgical Journal. In that communication Sir Brooke was disposed to place no reliance on any prophylactics; further experience only enables him to offer a few observations respecting oil friction and vaccination. As to the *former*:—

"There were so many instances of persons living in the closest intercourse with the infected *who escaped without the use of oil*, and so few well attested cases of persons having come into actual contact with pestiferous matter who *were protected by oil alone*, that I cannot hesitate to conclude that the opinion of its possessing any independent or certain prophylactic efficacy is destitute of foundation." 232.

The prophylactic powers of vaccination are not rated by our author in a higher degree than the oil frictions, and therefore we shall say nothing of it.

### TREATMENT.

In a disease of so multiform a character it was impossible, of course, to reduce the treatment to any thing like system; they were, therefore, obliged to confine their practice to obviating the most urgent symptoms. Our author adopted two principal indications:

"I. To moderate the symptoms denoting increased action of the arterial system, and especially those which marked considerable congestion in the brain.

"II. To sustain the powers of life when exhausted, and thereby to counteract putrescency." 235.

With the first intention he employed bleeding, emetics, laxatives, sudorifics, refrigerants, and blisters; and with the second, tonics and cordials. In respect to blood-letting, our author contented himself with leeching the temples; and even this measure he thinks was unproductive of much benefit—sometimes perhaps of mischief, by favouring the subsequent debility. He does not mean to assert, however, that bleeding is always inadmissible in plague. Of the other means Sir Brooke speaks very briefly, excepting mercury, on which so favourable a report was made by Mr. Stafford, and already given to the world. The latter gentleman states, that thirty or forty cases terminated favourably under his cure, when treated by this mineral.

The author had but two cases where he could fairly try the cold affusion, "and in one of them it was followed by decided and immediate amendment." The patient was almost instantaneously relieved from the distress of a dry, heated skin, and there succeeded a copious diaphoresis, with great abatement of the restlessness, and an inclination to sleep. A very remarkable instance is related, where the patient leapt twice into the sea, and speedily recovered. Upon the whole, our author thinks the remedy worthy of a trial, at the very beginning of the disease.

The work closes with a detail of cases under the author's own care in the Pest Hospital, and a copy of his examination before the Plague Committee of the House of Commons.

The perusal of this volume has left a strong impression on our minds in favour of the good sense, extensive erudition, correct judgment, and acute observation of its excellent author. We think too, that the facts and arguments brought forward by Sir Brooke Faulkner, must carry conviction, respecting the contagion of plague, to every mind not warped by the cloud of incomprehensible quiddities and cavils, which seems to have settled over the intellects of some of our brethren, and completely blinded them to the most plain and obvious facts, while they are straining their mental optics after invisible bodies which no where exist, except in the distempered imagination.

